# Response of microbial communities in the phyllosphere ecosystem of tobacco exposed to the broad-spectrum copper hydroxide

**DOI:** 10.3389/fmicb.2023.1229294

**Published:** 2023-09-28

**Authors:** Ruichao Feng, Hancheng Wang, Tingting Liu, Feng Wang, Liuti Cai, Xingjiang Chen, Songbai Zhang

**Affiliations:** ^1^MARA Key Laboratory of Sustainable Crop Production in the Middle Reaches of the Yangtze River (Co- construction by Ministry and Province), Yangtze University, Jingzhou, Hubei, China; ^2^Guizhou Provincial Academician Workstation of Microbiology and Health, Guizhou Academy of Tobacco Science, Guiyang, China

**Keywords:** phyllosphere ecosystem, copper hydroxide, high-throughput sequencing, biolog ECO metabolism, microbial community

## Abstract

Copper hydroxide is a broad-spectrum copper fungicide, which is often used to control crop fungal and bacterial diseases. In addition to controlling targeted pathogens, copper hydroxide may also affect other non-targeted microorganisms in the phyllosphere ecosystem. At four time points (before spraying, and 5, 10 and 15 days after fungicide application), the response of diseased and healthy tobacco phyllosphere microorganisms to copper hydroxide stress was studied by using Illumina high-throughput sequencing technology, and Biolog tools. The results showed that the microbiome communities of the healthy group were more affected than the disease group, and the fungal community was more sensitive than the bacterial community. The most common genera in the disease group were *Alternaria*, *Boeremia*, *Cladosporium*, *Pantoea*, *Ralstonia*, *Pseudomonas*, and *Sphingomonas*; while in the healthy group, these were *Alternaria*, *Cladosporium*, *Symmetrospora*, *Ralstonia*, and *Pantoea*. After spraying, the alpha diversity of the fungal community decreased at 5 days for both healthy and diseased groups, and then showed an increasing trend, with a significant increase at 15 days for the healthy group. The alpha diversity of bacterial community in healthy and diseased groups increased at 15 days, and the healthy group had a significant difference. The relative abundance of *Alternaria* and *Cladosporium* decreased while that of *Boeremia*, *Stagonosporopsis*, *Symmetrospora*, *Epicoccum* and *Phoma* increased in the fungal communities of healthy and diseased leaves. The relative abundance of *Pantoea* decreased first and then increased, while that of *Ralstonia*, *Pseudomonas* and *Sphingomonas* increased first and then decreased in the bacterial communities of healthy and diseased leaves. While copper hydroxide reduced the relative abundance of pathogenic fungi *Alternaria* and *Cladosporium*, it also resulted in the decrease of beneficial bacteria such as Actinomycetes and *Pantoea*, and the increase of potential pathogens such as *Boeremia* and *Stagonosporopsis*. After treatment with copper hydroxide, the metabolic capacity of the diseased group improved, while that of the healthy group was significantly suppressed, with a gradual recovery of metabolic activity as the application time extended. The results revealed changes in microbial community composition and metabolic function of healthy and diseased tobacco under copper hydroxide stress, providing a theoretical basis for future studies on microecological protection of phyllosphere.

## Introduction

Copper pollution in ecosystems has received considerable concerns in recent years. The main reason is that copper in the ecosystem is obviously enriched due to the extensive use of copper-based fungicides (Hirst et al., 1961; Santana Lima, 1994; Jacobson et al., 2005; Ballabio et al., 2018). Existing studies have shown that copper pollution has negative effects on soil microorganisms (Dumestre et al., 1999; Keiblinger et al., 2018; Carley et al., 2020; Peixoto et al., 2021), terrestrial invertebrates (Heikens et al., 2001; Lukkari and Haimi, 2005), aquatic life (Sun et al., 2016; Aksakal and Sisman, 2020) and plants (Gopalakrishnan Nair et al., 2014; AlQuraidi et al., 2019). Currently, research on the effects of copper on plants is focused on physiological, biochemistry, and toxicology aspects (Atha et al., 2012; Shi et al., 2013; Mosa et al., 2018; Rajput et al., 2018), but little has been done on the effects of copper on the phyllosphere ecosystem of tobacco. As a model crop for the study of phyllosphere ecosystem, tobacco has been extensively studied in recent years (Huang et al., 2021; Xiang et al., 2022).

The phyllosphere is the above-ground part of a plant that is colonized by microorganisms, including leaves, stems, flowers, and fruits (Lindow and Brandl, 2003; Knief et al., 2010). Phyllosphere microorganisms were exposed to relatively harsh environmental conditions with great variability for a long time, such as poor nutrition, lack of water resources, the release of antibacterial substances in leaves, intense ultraviolet irradiation, excessive temperature and humidity difference, and the existence of reactive oxygen species (Beattie and Lindow, 1999; Kadivar and Stapleton, 2003; Bashir et al., 2022). However, the phyllosphere microorganisms were still perform epiphytic or endophytic in the form of aggregate (Agler et al., 2016; Remus-Emserman and Schlechter, 2018). The community composition of phyllosphere microorganisms is highly diverse, including a variety of bacteria, archaea, fungi, algae, viruses, and not so frequent protozoas and nematodes (Lindow and Brandl, 2003; Inacio et al., 2010; Newton et al., 2010; Berlec, 2012). Bacteria are considered as the most abundant taxa of the phyllosphere, numerically averaging about 10^6^–10^8^ cells cm^−2^ of leaf tissue (Hirano and Upper, 2000; Remus-Emsermann et al., 2014). Phyllosphere microorganisms include both beneficial and pathogenic bacteria. Pathogenic fungi in tobacco are normally regard as microorganism that could induce plant diseases, such as *Alternara alternata* (Liu et al., 2022), *Rhizoctonia. Solani* (Sun et al., 2023). While beneficial bacteria are normally regarded as some microorganism that could protect the host against pathogen infection through antagonism, competition and induction of plant systemic resistance (Koutsoudis et al., 2006; Innerebner et al., 2011), such as *Pantoea* (Wright et al., 2001), *Pseudomonas* (De Meyer and Hofte, 1997), *Sphingomonas* (Khan et al., 2014). Phyllosphere beneficial microorganisms not only affect the biological cycle of carbon and nitrogen (Stanton et al., 2019; Abadi et al., 2021), but also have potential plant biogeography and ecosystem functions (Meyer and Leveau, 2012; Stone and Jackson, 2016), such as bioremediation of harmful chemicals and pollutants (Kuiper et al., 2004; Sandhu et al., 2007) and can produce plant hormones to promote plant growth (Reed et al., 2010; D’alessandro et al., 2014). In addition, overgrowth of interfoliar pathogens or loss of microbial diversity can also lead to ecological imbalance and plant disease (Chen Q. et al., 2020). Changes in microbial communities are often the precursor of changes in the health and viability of the whole environment. Studying microbial changes can predict the changing trend of the phyllosphere ecosystem.

The widespread use of chemical pesticides may cause specific off-target effects of organisms, resulting in ecological imbalance of microbial environment (Blacquière et al., 2012). For example, the insecticide cypermethrin influences the phyllosphere community composition of cucumber by increasing the total number of bacteria and decreasing the number of fungi (Zhang et al., 2008). The application of the broad spectrum fungicide N-(3,5-dichlorophenyl) succinimide had a greater effect on various rare microorganisms in the tobacco phyllosphere (Chen et al., 2021). The study of Cernava et al. (2019) showed chemical (lime sulfur) after chemical (lime-sulfur) treatment, the bacterial community of plants increased by 11 taxa. The application of fungicide enostroburin resulted in significant changes in the composition of the phyllosphere bacterial community in wheat, among which the abundance of *Pantoea* sp. community increased significantly (Gu et al., 2010). Perazzolli et al. (2014) also found a chemical fungicide based on sterol biosynthesis inhibition (penconazole) treatment reduced the abundance of deltaproteobacteria of UD populations.

As a broad-spectrum fungicide, copper hydroxide has been used to control fungal and bacterial diseases of peanut (*Arachis hypogaea* L.), olive tree (*Canarium oleosum*), pear (*Pyrus spp*), tomato (*Solanum lycopersicum* L.), *Allium cepa* (*Allium cepa* L.), pepper (*Piper nigrum* L.) and other crops (Culbreath et al., 1992; Buonaurio et al., 2002; Gent and Schwartz, 2005; Soares et al., 2006; Yu et al., 2009; Abbasi and Weselowski, 2015). The study of Zhang et al. (2021) showed ultrathin Ni(OH)_2_ nanosheets decorated with amorphous Cu(OH)_2_ islands are identified to be a highly efficient catalyst for the selective electrooxidation of HMF. The study of Belal et al. (2021) showed Cu(OH)_2_ nanorods can be used as a stable oil–water separation material by deposition. The mode action mechanism of this compound is that the released copper ions interact with the -SH, -NH, -OH and other groups in the enzymes and proteins of bacteria or fungi, resulting in enzyme deactivation and denateration, inhibiting microbial metabolism and reproduction, and thus achieving bactericidal effect (Gisi and Sierotzki, 2008). Non-specific fungicides have the potential to cause changes in the composition of non-target microorganisms in the leaf circle, including those that may be beneficial to the plant (Berlec, 2012). Studies have shown that copper hydroxide can affect soil microbial community and lead to changes in soil microbial composition (Peixoto et al., 2021). Exposure to copper hydroxide induced changes in metabolites of lettuce, cucumber, corn and spinach leaves (Zhao et al., 2016, 2017a,b,c, 2018, 2020). However, the effect of copper hydroxide on the phyllosphere microbial community of tobacco has not been studied. In addition, the metabolic function of copper hydroxide on tobacco phyllosphere microorganisms was not well understood.

Therefore, the objectives of this study were as follows: (i) to compare the microbial community composition and diversity of healthy leaves and disease leaves under copper hydroxide stress; (ii) to compare the effects of copper hydroxide on phyllosphere microbial metabolism between healthy leaves and disease leaves. Through this study, we hope to understand the natural dynamic response of phyllosphere microorganisms to copper stress and understand the impact of copper hydroxide on phyllosphere ecosystems, so as to provide theoretical basis for future research on the response of phyllosphere microorganisms to pesticide application.

## Materials and methods

### Sampling sites and sampling strategy

In August 2020, a test was conducted on a Yunyan 105 tobacco field infected with tobacco brown spot caused by *Alternaria alternata* was used for the experiment in the area of Heishou Town, Weining County, Bijie City, Guizhou Province (26.74 N, 104.02E) of China. Before the experiment, tobacco brown spots appeared sporadously on tobacco plants (Yunyan 105) in the field, and the incidence rate was about 15–25%. The fully randomized block was designed to subject plants in an open field, when tobacco brown spot occurs sporadically in the field, treated with 57.6% Cu(OH)_2_ WP (Newham, Qingdao, China), and the dosage is the recommended dosage (0.52 a.i. kg/ha). A multi-function sprayer (model: DSF01A-20-100, Guizhou Qian Fengyuan Agricultural Technology Development Co., Ltd.) was used to evenly spray the leaf surface until the droplet was lost.

At 0 days (before treatment) and then 5, 10, and 15 days, the tobacco leaf tissues of diseased and healthy plants that had been treated with copper hydroxide were cut with sterilizing scissors. Three biological replicates were conducted. Leaf samples were placed in sterile 50 mL centrifuge tubes and stored at 4°C in a cryopreservation box. One part was used for metabolic function study (5 g), and the other part was stored in the refrigerator at −80°C for application sequencing.

For designating codes to samples, the first and second letters QY mean copper hydroxide, the next letter J was used for healthy tobacco, and B for diseased tobacco leaves, followed by a number, which represents the time taken for samples to be taken, 0 for the 0 days before application, 1 for 5 days after application, 2 for 10 days after application, 3 for 15 days, and then followed by 1 for the first replicate, 2 for the second replicate, 3 for the third replicate. To be more specific, QYB33 represents the third replicate of the diseased leaves after 15 days of copper hydroxide application (Table 1). In this study, healthy and diseased tobacco leaves were selected to study the effects of copper hydroxide on phyllosphere microbial community composition and metabolism before the application of fungicide and 5, 10, 15 days after the application of fungicide.

**Table 1:** Sample collection information.

Sampling		0 Day before application	5 Days after application	10 Days after application	15 Days after application
Healthy tissue	Sample no.	QYJ01	QYJ11	QYJ21	QYJ31
		QYJ02	QYJ12	QYJ22	QYJ32
		QYJ03	QYJ13	QYJ23	QYJ33
	Group no.	QYJ0	QYJ1	QYJ2	QYJ3
Diseased tissue	Sample no.	QYB01	QYB11	QYB21	QYB31
		QYB02	QYB12	QYB22	QYB32
		QYB03	QYB13	QYB23	QYB33
	Group no.	QYB0	QYB1	QYB2	QYB3

### DNA extraction, PCR amplification, and high-throughput sequencing

Total DNA of tobacco samples was extracted by CTAB method (Saghai-Maroof et al., 1984). DNA concentration and purity were determined by 1% agarose gel electrophoresis. According to the concentration, DNA was diluted to 1 ng/μL using sterile water. The fungal primers ITS1-5F-F (5’-GGAAGTAAAAGTCGTAACAAGG-3′) and ITS1-1F-R (5’-GCTGCGTTCTTCATCGATGC-3′) were used as templates to amplify the fungal ITS region (Zhang et al., 2010). Bacterial primers 515F (5’-GTGYCAGCMGCCGCGGTAA-3′) and 806R (5’-GGACTACHVGGGTWTCTAAT-3′) were used to amplify the V4 region of bacteria (Thompson et al., 2017). The PCR reaction consisted of 20 μL (4 μL 5 × FastPfu Buffer, 2 μL 2.5 mM dNTPs, 0.8 μL each primer, 0.4 μL FastPfu polymerase, and 1 μL DNA and 11.8 μL water). Fungal and bacterial PCR reactions were performed on a peqSTAR thermocycler (PEQLAB Ltd., United Kingdom). Fungal PCR was set up as follows: 94°C for 5 min, followed by 35 cycles of 94°C for 1 min, 57°C for 1 min, 72°C for 1 min, and finally at 72°C for 5 min. The bacterial PCR setup consisted of the following steps: 94°C for 3 min, followed by 30 cycles of 94°C for 45 s, 55°C for 45 s, 72°C for 90 s, and finally 72°C for 7 min (Huang et al., 2021). PCR products were checked by 2% agarose gel electrophoresis and purified with Gene JET (Thermo Fisher Scientific, Waltham, MA, United States), then sent for sequencing (250 bp pairedend sequencing) according to the standard protocol. The IonS5 XL platform (Thermo Fisher Scientific, Waltham, MA, United States) was used for high-throughput sequencing at Novogene Bioinformatics Technology Co., Beijing, China.

### Effect of copper hydroxide on the metabolic function of leaf microorganisms in tobacco leaves

One gram of diseased and healthy mixed tobacco leaves at different periods were taken and placed in a 100 mL triangular flask containing 50 mL 0.8% sterile normal saline. The flask was incubated at 180 rpm for 2 h at 28°C, and then stood for 30 min at night after shaking. 100 μL of the supernatant was added to the test well of Biolog ECO plate in turn (Grove et al., 2004). The ECO plate was placed in OmniLog incubator and incubated at 28°C for 7 days. Biolog D5E_OKA_data.exe software was used to collect the color changes in the metabolic wells of tobacco phyllosphere microorganisms during the growth process. After collection, HemI software was used to make a heat map to analyze the metabolic function of sample interfoliar microorganisms (Deng et al., 2014).

### Data processing

The sequencing data were spliced using FLASH and fastp software, and the chimeric sequences were filtered and removed to obtain the final Effective Tags. UPARSE software (Version 7.1) was used to cluster all Effective Tags of all samples into OTUs (operational taxa) with 97% similarity, and representative OTUs sequences were selected for species annotation (Edgar, 2013). The OTUs of fungal ITS were annotated using Qiime software (Version 1.9.1) and Unite (v8.2) database (Altschul et al., 1990; Kõljalg et al., 2013). The OTUs of Bacterial 16S rRNA were annotated using the SSUrRNA database of SILVA138.1(Wang et al., 2007; Quast et al., 2012). Fast multiple sequence alignment was performed using MUSCLE (Version 3.8.31) software to obtain phylogenetic relationships of all OTUs representative sequences (Manuel, 2013). Finally, microbial community diversity (Shannon and Simpson index), richness (Chao1 index), Goods Coverage index and beta diversity were estimated by Qiime software (Version 1.9.1) for different time series samples. Shannon and Simpson index was used to analyze the microbial community diversity of tobacco leaves. Chao 1 index and ACE index were used to analyze the community abundance in community samples (Zhang et al., 2019). Goods Coverage index was used to analyze the microbial community coverage of samples (Xie et al., 2021). PCoA and PCA were used to determine differences between community structures. Samples with higher community similarity tended to cluster together. Fungal trophic patterns were analyzed by FUNGuild database (Nguyen et al., 2016). Bacterial metabolic functions were analyzed by PICRUSt database (Langille et al., 2013). Cytoscape 3.9.1 software was used to assess the interactions between microbial communities (Williams et al., 2014; Meng et al., 2017). R software (Version 2.15.3) was used to draw the dilution curve, Rank abundance curve and species accumulation curve.

### Statistical analysis

IBM SPSS Statistics 23 (IBM Corp., New York, United States) was used to compare the differences in alpha-diversity indexes between fungal and bacterial communities (Mao et al., 2020). The mean values were compared, and *p* ≤ 0.05 was considered statistically significant.

## Results

### Quality of bacterial and fungal sequence data

**Figure 1:**
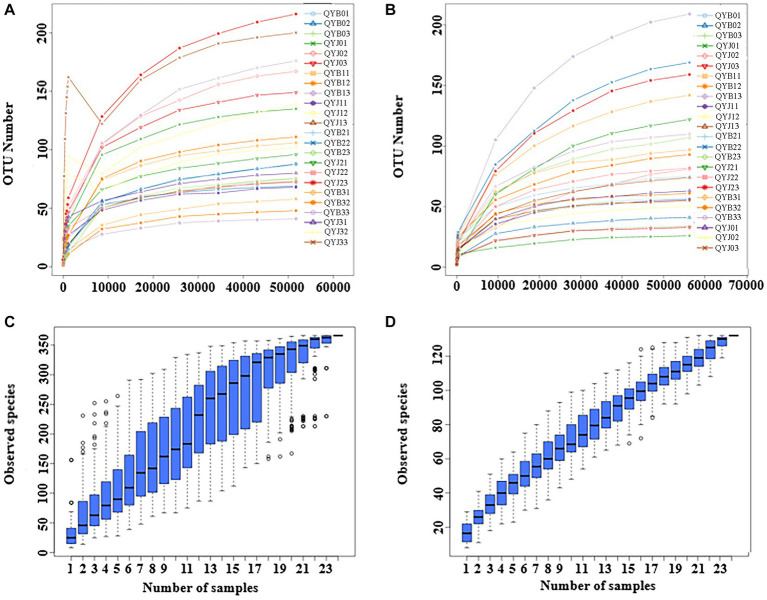
Rarefaction curves of fungal **(A)** and bacterial **(B)** OTUs across different tobacco leaf samples. Species accumulation boxplot of fungal **(C)** and bacterial **(D)** OTUs in different leaf samples. (In the dilution curve, the horizontal coordinate is the number of sequencing strips randomly selected from a sample, and the vertical coordinate is the number of OTUs that can be built based on the number of sequencing strips, which is used to reflect the sequencing depth. Different samples are represented by curves of different colors).

After quality control processing, denoising and chimeric filtering, a total of 1,557,233 fungal sequences and 1,557,862 bacterial sequences were obtained from 24 samples. At 97% similarity level, the fungal sequences were classified into 623 operational taxa (OTUs), and the bacterial sequences were classified into 773 OTUs. When the sequence number of fungi and bacteria reached 50,000 and 60,000, respectively, all sparse curves reached the plateau stage (Figures 1A,B), suggesting that sequencing depth could reflect community structure and diversity. When the Number of samples reached 24, the position of the box chart tended to be flat, indicating sufficient sampling and subsequent data analysis could be carried out (Figures 1C,D).

### Change of diversity of healthy and diseased tobacco leaves a under copper hydroxide stress

Diversity (Shannon and Simpson) index, richness (Chao1) index and goods coverage index were used to analyze the fungal and bacterial OTU diversity in the infected and healthy groups at different application periods. The fungal coverage index for healthy and infected tobacco samples was above 0.96 and the bacterial coverage index was above 0.85. It indicated that the sequence detection probability of the samples is high, and the sequencing results can represent the actual microbial community structure in the samples (Table 2).

**Table 2:** Effect of copper hydroxide on the alpha diversity of tobacco phyllosphere microorganisms.

	Application time	Group	Shannon	Simpson	Chao 1	Goods coverage
Fungi	0 Day	QYB0	0.93 ± 0.42bc	0.25 ± 0.14bc	19.69 ± 4.16a	0.995 ± 0.002a
		QYJO	2.83 ± 0.17ab	0.76 ± 0.03ab	68.4 ± 6.63a	0.982 ± 0.002a
	5 Days	QYB1	0.36 ± 0.08c	0.09 ± 0.02c	14.78 ± 2.90a	0.995 ± 0.001a
		QYJ1	2.19 ± 0.89abc	0.57 ± 0.19abc	61.02 ± 32.45	0.981 ± 0.011a
	10 Days	QYB2	0.55 ± 0.09bc	0.15 ± 0.04c	33.78 ± 18.60a	0.992 ± 0.003a
		QYJ2	2.32 ± 0.49abc	0.59 ± 0.12abc	60.31 ± 12.43a	0.981 ± 0.004a
	15 Days	QYB3	1.41 ± 0.35bc	0.41 ± 0.12abc	42.8 ± 9.54a	0.986 ± 0.003a
		QYJ3	4.33 ± 0.72a	0.89 ± 0.03a	111.54 ± 41.44a	0.968 ± 0.013a
Bacteria	0 Day	QYB0	2.02 ± 0.64bc	0.57 ± 0.16b	15.33 ± 3.71a	0.935 ± 0.015a
		QYJO	1.17 ± 0.17c	0.32 ± 0.04c	16 ± 3.79a	0.926 ± 0.023a
	5 Days	QYB1	2.88 ± 0.34ab	0.79 ± 0.05ab	35.17 ± 9.33a	0.883 ± 0.023a
		QYJ1	2.55 ± 0.47ab	0.67 ± 0.12ab	25.42 ± 3.55a	0.892 ± 0.016a
	10 Days	QYB2	3.08 ± 0.38ab	0.79 ± 0.04ab	39.44 ± 16.52a	0.866 ± 0.046a
		QYJ2	2.17 ± 0.33abc	0.57 ± 0.09b	36.11 ± 4.70a	0.853 ± 0.012a
	15 Days	QYB3	2.68 ± 0.17ab	0.76 ± 0.03ab	24.67 ± 8.19a	0.922 ± 0.013a
		QYJ3	3.13 ± 0.24a	0.82 ± 0.03a	25 ± 2.02a	0.896 ± 0.015a

Data followed by the same letter in the same line are not significantly different by the Tukey test in SPSS (*p* > 0.05).

**Figure 2:**
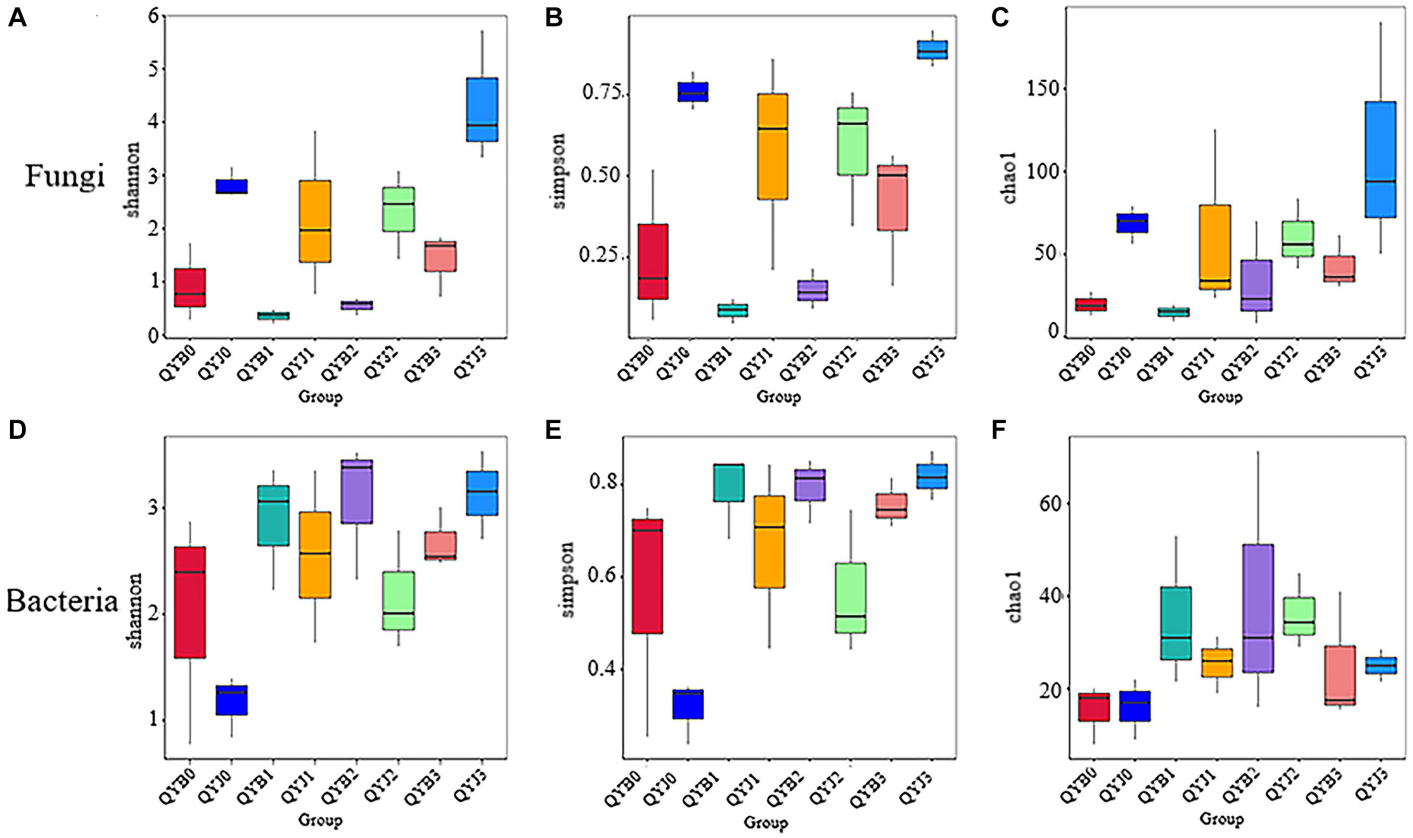
Shannon **(A)**, simpson **(B)**, chao1 **(C)** Alpha diversity index of fungi in different leaf samples. Shannon **(D)**, simpson **(E)**, chao1 **(F)** Alpha diversity index in bacterial samples from different tobacco leaves (in the analysis of inter-group difference of Alpha diversity index, the box chart can directly reflect the median, dispersion, maximum, minimum and outlier values of species diversity within the group. At the same time, Tukey test was used to analyze whether the differences in species diversity between groups were significant).

Among the fungal OTUs, the diversity index and richness index of healthy tobacco leaves were higher than those of diseased leaves before and after the application of the fungicide, but the diversity indices of healthy and diseased leaves were significantly different after applying the fungicide only on day 15, at 4.33 and 1.41, respectively. After 5 days of fungicide application, the diversity and richness of healthy and diseased tobacco leaves decreased to varying degrees. After that, the diversity and richness of healthy tobacco leaves and diseased tobacco leaves continued to rise (Figures 2A–C).

Among the bacterial OTUs, the diversity index of healthy tobacco leaves before application of fungicide was lower than that of diseased leaves, while the richness index was higher than that of diseased leaves, but there was no significant difference. After 5 days of fungicide application, the diversity and richness of healthy and diseased tobacco leaves increased to varying degrees. After 15 days of fungicide application, the diversity index of healthy tobacco leaves was higher than that of diseased leaves, which might be due to the greater effect of copper hydroxide on healthy leaves (Figures 2D–F).

### Changes of fungal community composition in healthy and diseased tobacco leaves under copper hydroxide stress

**Figure 3:**
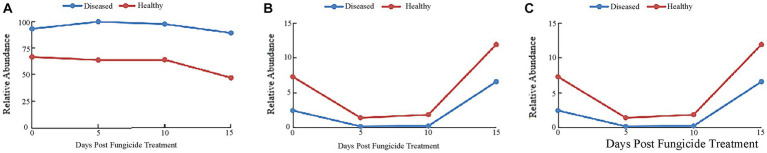
Changes in fungal community compositions at the phylum level for diseased and healthy leaves over time after copper hydroxide. **(A)** Ascomycota, **(B)** Basidiomycota, **(C)** other. The “other” group includes all organisms that are not recognized or have a relative abundance of <0.1%.

The ITS dataset showed that 48.93% of OTUs were mainly divided into Ascomycota and Basidiomycota. The “other” group in Figure 3 includes all organisms that are not recognized or have a relative abundance of <0.1%. Before copper hydroxide treatment, the phyllosphere microorganisms of healthy and disease tobacco leaves were mainly divided into Ascomycota (66.52 and 92.85%) and Basidiomycota (7.30 and 2.47%). With the application of copper hydroxide, the abundance of Ascomycota in diseased leaves changed little over time, but the abundance of Ascomycota in healthy leaves was reduced by 19.59% compared to before application (Figure 3A). The relative abundance of Basidiomycota in healthy and diseased leaves decreased by 5.88 and 2.28% at 5 days after application, and thereafter increased by 10.4 and 6.4% at 15 days after application (Figure 3B). The “other” group showed a similar pattern in healthy and diseased leaves, with abundance declining at 5 days and peaking at 15 days., when the relative abundance of healthy leaves was 40.04% and diseased leaves only 4.42% (Figure 3C).

**Figure 4:**
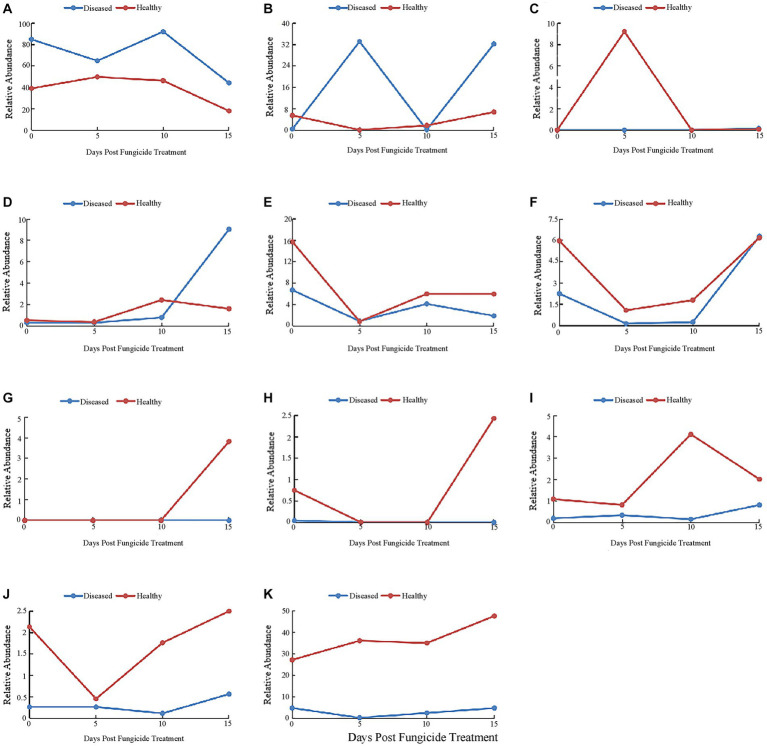
Changes in fungal community compositions at the genus level for diseased and healthy leaves over time after copper hydroxide. **(A)** *Alternaria*, **(B)** *Boeremia*, **(C)** *Issatchenkia*, **(D)** *Stagonosporopsis*, **(E)** *Cladosporium*, **(F)** *Symmetrospora*, **(G)** *Pseudeurotium*, **(H)** *Thanatephorus*, **(I)** *Epicoccum*, **(J)** *Phoma*, **(K)** other. The “other” group includes all organisms that are not recognized or have a relative abundance of <0.1%.

At the genus level, the 10 most common fungal genera are shown in Figure 4. The main genera of healthy tobacco leaves and diseased tobacco leaves before fungicide application were *Alternaria* (38.91 and 84.79%), *Boeremia* (5.47 and 0.41%), *Cladosporium* (15.69 and 6.67%), *Symmetrospora* (5.96 and 2.25%), *Epicoccum* (1.09 and 0.19%), and *Phoma* (2.13 and 0.26%). After the application of copper hydroxide, *Alternaria* in healthy and diseased leaves showed a general downward trend, decreasing by 21.16 and 40.82%, respectively, at 15 days (Figure 4A). The relative abundance of *Boeremia* did not change much with time in healthy leaves, but increased significantly in diseased leaves and reached a peak of 33.11% at 5 days (Figure 4B). *Issatchenkia* was not detected in diseased leaves and reached a peak of 9.21% in healthy leaves at 5 days (Figure 4C). The relative abundance of *Stagonosporopsis* in healthy leaves changed little over time and reached a peak of 9.03% at 15 days in diseased leaves (Figure 4D). The relative abundance of *Cladosporium* showed a similar pattern in healthy and diseased leaves, decreasing by 14.77 and 5.81% at 5 days, respectively (Figure 4E). The relative abundance of *Symmetrospora* showed similar trends in healthy and diseased leaves, decreasing by 4.87 and 2.1% at 5 days, respectively (Figure 4F). *Pseudeurotium* was not detected in diseased leaves and reached a peak of 3.82% at 15 days in healthy leaves (Figure 4G). *Thanatephorus* was also not detected in diseased leaves, but was detected in healthy leaves at 0 days and 15 days, peaking at 2.43% on 15 days (Figure 4H). *Epicoccum* reached a peak of 4.12% at 10 days in healthy leaves, but changed little over time in diseased leaves (Figure 4I). *Phoma* also showed little change over time in diseased leaves, while peaking at 2.51% at 15 days in healthy leaves (Figure 4J). The “other” group showed little change over time in diseased leaves but reached a peak of 47.64% at 15 days in healthy leaves (Figure 4K).

### Changes of bacterial community composition in healthy and diseased tobacco leaves under copper hydroxide stress

**Figure 5:**
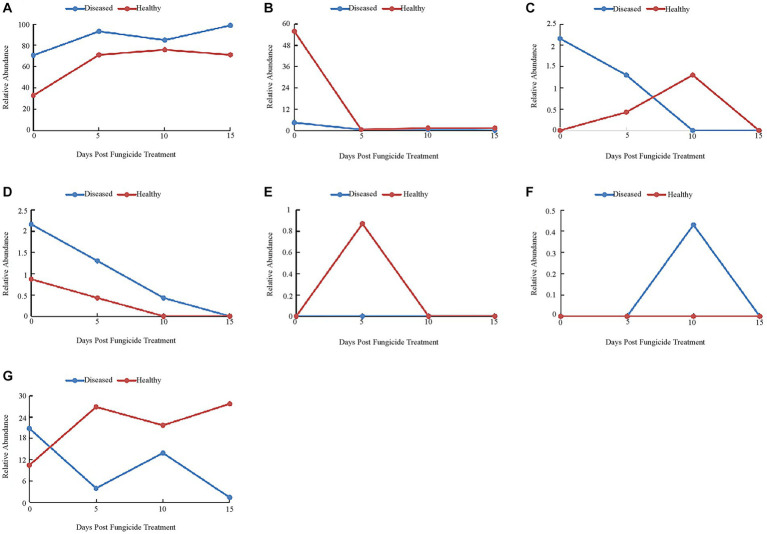
Changes in bacterial community compositions at the phylum level for diseased and healthy leaves over time after copper hydroxide. **(A)** Proteobacteria, **(B)** Firmicutes, **(C)** Bacteroidetes, **(D)** Actinobacteria, **(E)** Fusobacteria, **(F)** unidentified_Bacteria, **(G)** other. The “other” group includes all organisms that are not recognized or have a relative abundance of <0.1%.

The 16S dataset showed that 37.28% of OTUs were mainly divided into Proteobacteria, Firmicutes, Bacteroidetes, Actinobacteria and Fusobacteria. Before copper hydroxide treatment, the phyllosphere microorganisms of healthy and disease tobacco leaves were mainly divided into Proteobacteria (32.9 and 70.56%) and Firmicutes (55.84 and 4.33%) (Figure 5). With the application of copper hydroxide, the relative abundance of Proteobacteria and Firmicutes showed similar patterns in healthy and diseased leaves. On the fifth day, Proteobacteria increased by 38.1 and 28.14%, while Firmicutes decreased by 54.54 and 4.33%, respectively (Figures 5A,B). The relative abundance of Bacteroidetes had no obvious pattern, but was not detected at 15 days (Figure 5C). The relative abundance of Actinobacteria showed a similar pattern in healthy leaves and diseased leaves, decreasing by 0.87 and 2.16%, respectively, at 15 days, when Actinobacteria could not be detected (Figure 5D). Fusobacteria was only detected in healthy leaves at 5 days, while unidentified Bacteria was only detected in diseased leaves at 10 days (Figures 5E,F). The “other” group showed the opposite pattern in healthy and diseased leaves, with a 17.32% increase in abundance in healthy leaves and a 19.43% decrease in abundance in diseased leaves at 15 days (Figure 5G).

**Figure 6:**
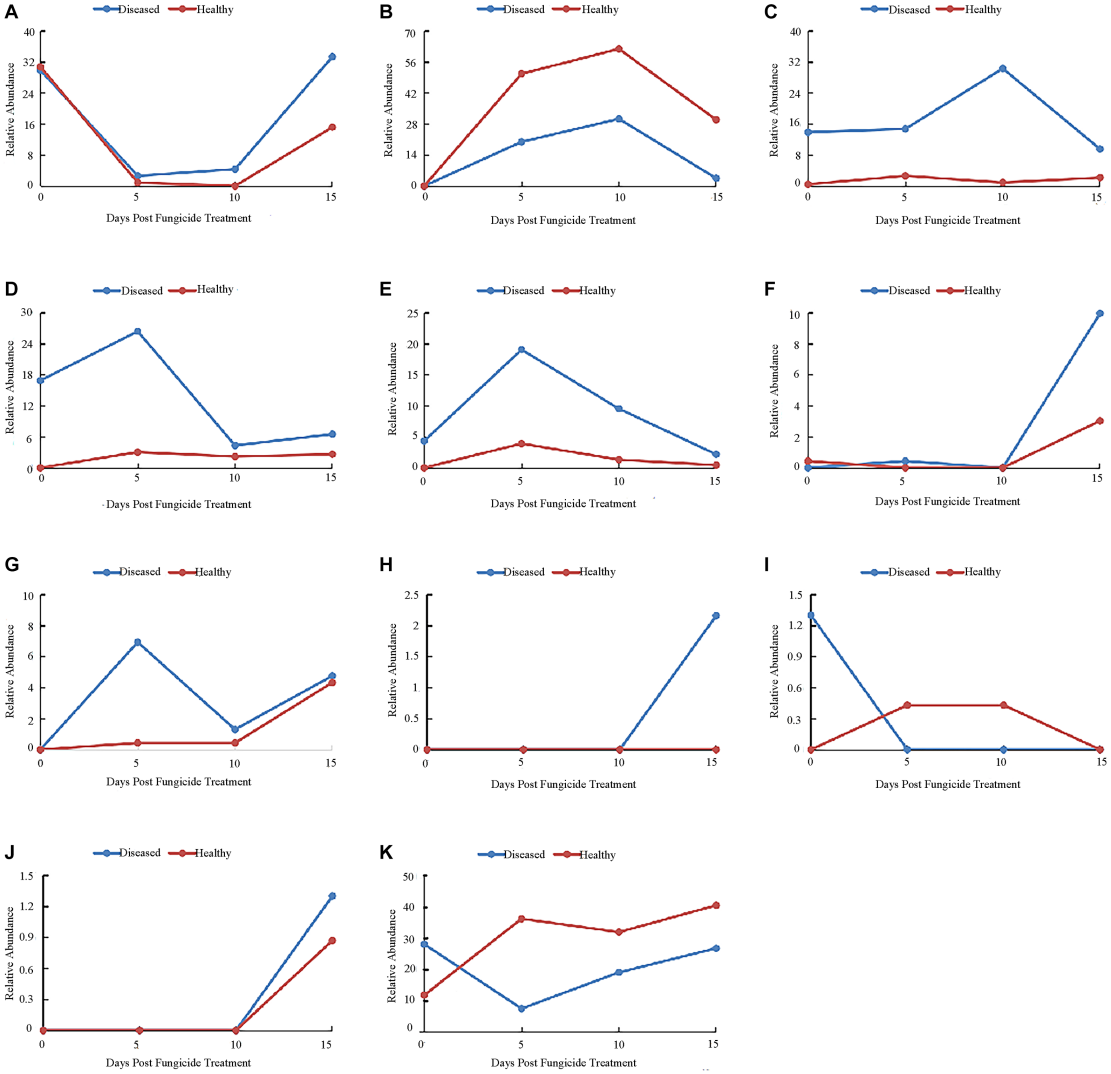
Changes in bacterial community compositions at the genus level for diseased and healthy leaves over time after Copper hydroxide. **(A)** *Pantoea*, **(B)** *Ralstonia*, **(C)** *Pseudomonas*, **(D)** *Sphingomonas*, **(E)** *Massilia*, **(F)** *Erwinia*, **(G)** *Methylobacterium*, **(H)** unidentified-Enterobacteriaceae, **(I)** *Bacteroides*, **(J)** *Serratia*, **(K)** other. The “other” group includes all organisms that are not recognized or have a relative abundance of <0.1%.

At the genus level, the 10 most common bacterial genera are shown in Figure 6. Before copper hydroxide treatment, the phyllosphere microorganisms of healthy and disease tobacco leaves were mainly divided into *Pantoea* (30.74 and 29.87%), *Pseudomonas* (0.43 and 13.85%) and *Sphingomonas* (0 and 16.88%). After the application of copper hydroxide, the relative abundance of *Pantoea* showed a similar pattern in healthy and diseased leaves, decreasing by 29.87 and 27.27% at 5 days, respectively (Figure 6A). The relative abundance of *Ralstonia* peaked at 61.9 and 30.30% in both healthy and diseased leaves at 10 days (Figure 6B). The relative abundance of *Pseudomonas* did not change with time in healthy leaves but reached a peak of 30.30% at 10 days in diseased leaves (Figure 6C). The relative abundance of *Sphingomonas* changed little over time in healthy leaves and peaked at 26.41% at 5 days in diseased leaves (Figure 6D). The relative abundance of *Massilia* did not change with time in healthy leaves but reached a peak of 19.05% at 5 days in diseased leaves (Figure 6E). The relative abundance of *Erwinia* peaked at 3.03 and 9.96% in healthy and diseased leaves at 15 days (Figure 6F). The relative abundance of *Methylobacterium* was not detected in both healthy and diseased leaves at 0 days, and reached a peak of 4.33% at 15 days in healthy leaves and 6.93% at 5 days in diseased leaves (Figure 6G). unidentified *Enterobacteriaceae* was not detected in healthy leaves but reached a peak of 2.16% in diseased leaves at 15 days (Figure 6H). *Bacteroides* was detectable in diseased tobacco leaves only at 0 days, but was detectable in healthy tobacco leaves at 5 and 10 days (Figure 6I). *Serratia* was detected only in healthy leaves and diseased leaves at 15 days, when the peak values were 0.87 and 1.30% (Figure 6J). The relative abundance of The “other” group did not change much over time in diseased leaves, but reached a peak of 40.69% at 15 days in healthy leaves (Figure 4K).

### Distribution of bacterial and fungal and communities between leaves

**Figure 7:**
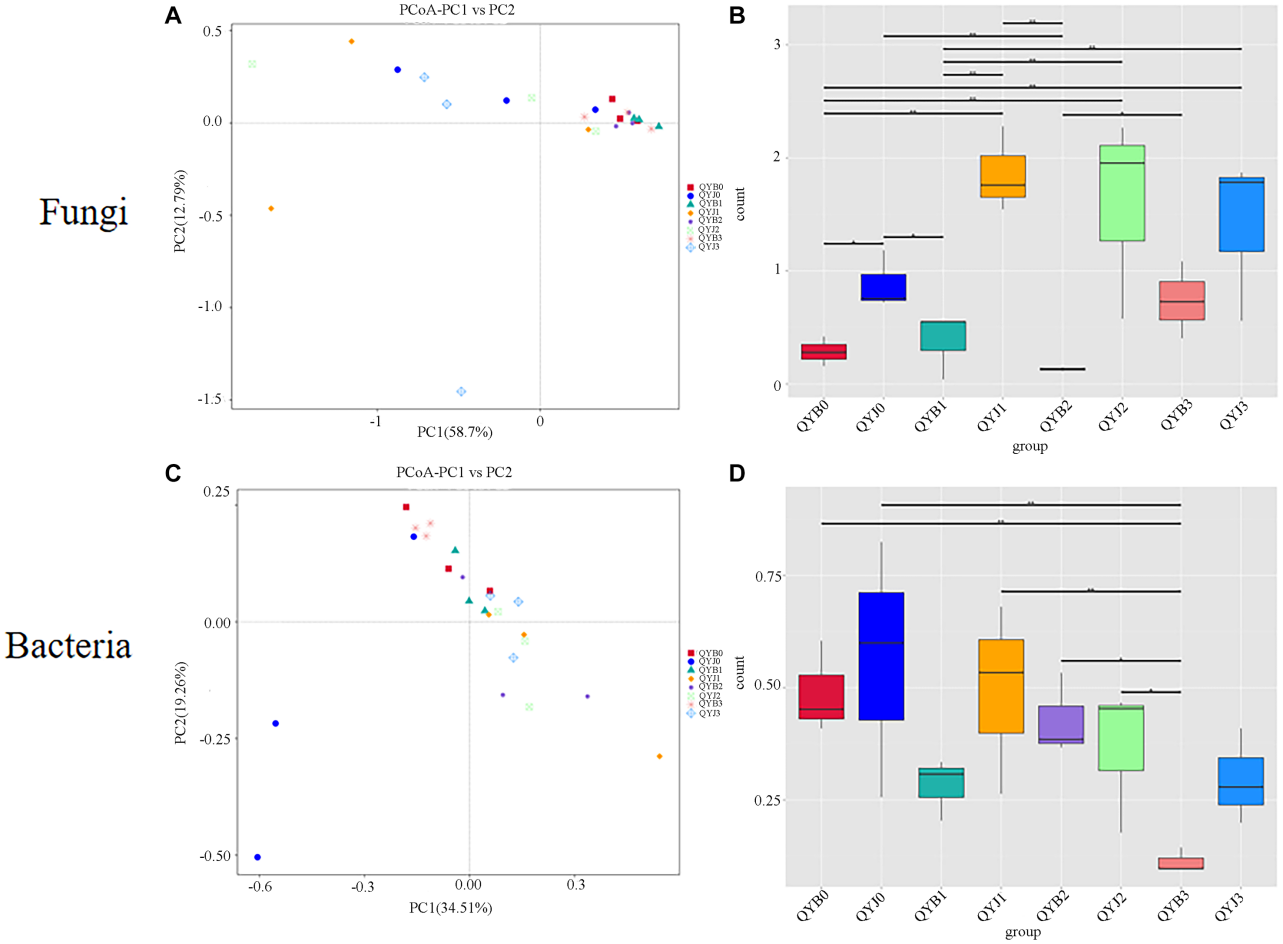
Principal co-ordinate analysis (PCoA) analysis of the fungal **(A)** and bacterial **(C)** communities in the different group samples. Group difference analysis of fungal beta diversity index in different tobacco samples **(B)** and group difference analysis of bacterial beta diversity index **(D)**.

Principal Co-ordinates Analysis (PCoA) plots were used to determine the spatial distribution of fungal and bacterial communities (Figures 7A,C). In the fungal community, all diseased groups tended to cluster together, suggesting that copper hydroxide has less effect on the phyllosphere microorganism of diseased samples. However, the dispersion degree among healthy samples is large, indicating that copper hydroxide has a great influence on healthy groups (Figure 7B). In the bacterial community, the samples of the diseased group and the healthy group were more discrete (Figure 7D).

### Changes of functional characteristics of healthy and diseased tobacco leaves under copper hydroxide stress

**Figure 8:**
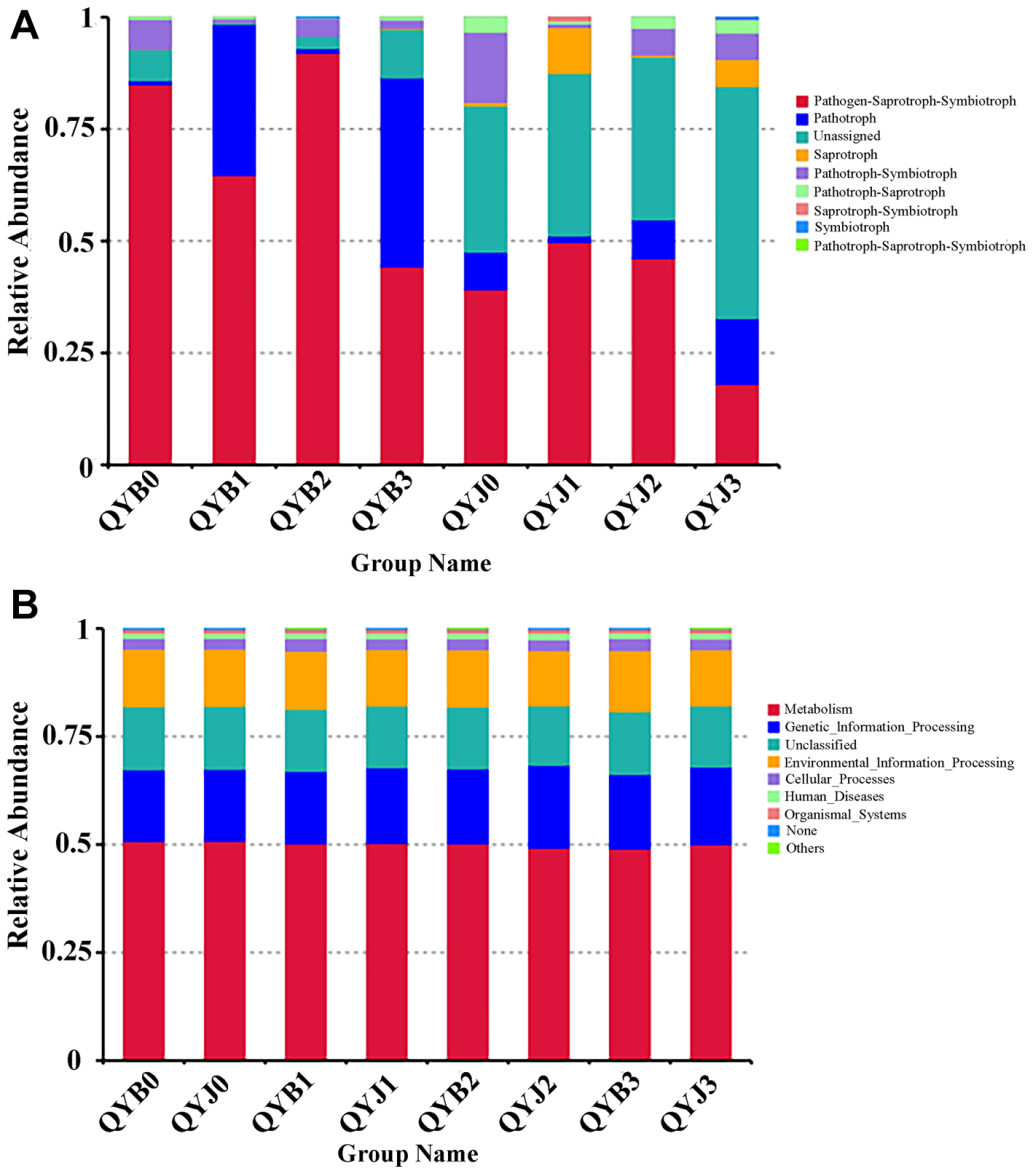
Relative abundance of fungal **(A)** and bacterial **(B)** functional groups (guilds) based on OTU annotation table with disturbance frequency level (according to the database annotation results, the functional abundance results of relevant aspects were counted).

The FUNGuild database was used to analyze the ecological function of the fungi in the samples in this study (Figure 8A). Fungal OTUs can be subdivided into nine groups according to nutrient patterns. Before the application of copper hydroxide, pathogen-saprophytic-symbiotic (84.94%) and pathogen-symbiotic (6.67%) were the dominant ecological functions of fungi in the disease group. The fungal dominant ecological functions of the healthy group were Pathotroph-Saprotroph-Symbiotroph (39.14%), Pathotroph-Symbiotroph (15.69%), Pathotroph (8.39%), Pathotroph-Saprotroph (3.30%) and Unassigned (32.66%). After treatment with copper hydroxide, the prediction of ecological function of fungi was changed. With the increase of time, the relative abundance of pathotroph-Saprotroph-Symbiotroph and Pathotroph-Symbiotroph decreased by 40.71 and 4.83%, respectively, in the diseased group, while the relative abundance of Pathotroph increased by 41.31%. The relative abundance of Pathotroph, Saprotroph and Unassigned increased by 6.25, 5.25 and 19.18%, respectively, in the healthy group. Meanwhile, the relative abundance of Pathotroph-Saprotroph-Symbiotroph and Pathotroph-Symbiotroph decreased by 21.09 and 9.77%, respectively.

PICRUSt for functional prediction of KEGG database based on 16S sequencing data, The results showed that 24 samples from 4 different periods contained 6 known biological metabolic pathways at the primary functional level (Figure 8B): “environmental information processing,” “cellular processes,” “genetic information processing,” “human diseases,” “metabolism” and “organic systems.” For the prediction of bacterial function, the gene sequences of the diseased and healthy groups were mainly related to “metabolic” function, followed by “genetic information processing” and “environmental information processing.” Copper hydroxide treatment had little effect on the prediction of bacterial function.

### Metabolic changes of healthy and diseased tobacco leaves under copper hydroxide stress

**Figure 9:**
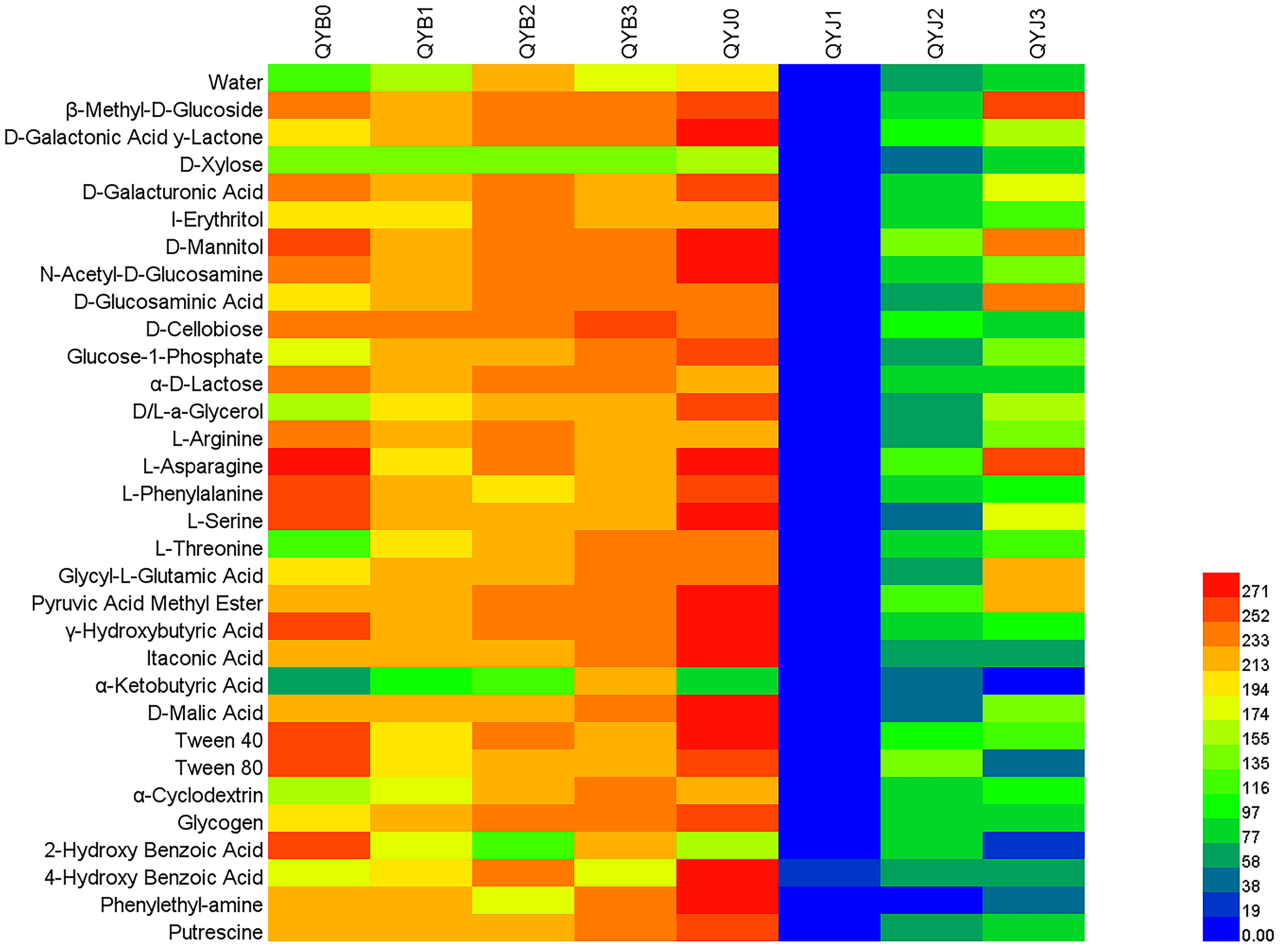
Cluster heat map of the effect of copper hydroxide on metabolism of tobacco phyllosphere microorganism. The color values represent the degree of the microbes metabolizing carbon sources. The value 0–100, 100–200 and > 200 represent the leaf microbes metabolized in Biolog ECO microplate poorly, moderately, effectively, respectively.

Biolog ECO plates contain carbon sources used by most microorganisms in nature. There are 31 carbon sources, including carbohydrates, carboxylic acids, amino acids, phenolic acids, polymers and amines. The effects of copper hydroxide application on phyllosphere microbial metabolic function were significantly different between the diseased and healthy groups (Figure 9). Before the application of copper hydroxide, the diseased group microorganisms could efficiently metabolize 24 carbon sources, while the metabolic capacity of D-xylose, glucose-1-phosphate, D, L-a-glycerol, L-threonine, α-ketobutyric Acid, α-cyclodextrin and 4-hydroxybenzoic Acid was weak. Healthy group microorganisms can efficiently metabolize 28 carbon sources in addition to D-Xylose, 2-Hydroxy Benzoic Acid and α-Ketobutyric Acid.

The metabolic function of the diseased group was greatly affected after the application of fungicide. After 5 days of copper hydroxide application, the phyllosphere microorganisms in the diseased group increased the metabolism of 11 carbon sources, including D-galactolactone, I-erythritol, 4-hydroxybenzoic Acid, α-cyclodextrin, L-threonine, glycogen, D-glucosamine, Glycine-L-glutamic Acid, Glucose-1-phosphate, α-ketobutyric Acid, D, L-a-glycerol. The microorganisms in the diseased group can efficiently metabolize 29 carbon sources in addition to D-Xylose, 2-Hydroxy Benzoic Acid and α-Ketobutyric Acid. After 10 days of copper hydroxide application, the metabolism capacity of the phyllosphere microorganisms in the diseased group was further improved except for the three carbon sources of 2-Hydroxy Benzoic Acid, L-Phenylalanine and Phenylethyl-amine. After 15 days of copper hydroxide application, the metabolic capacity of the diseased group was further improved, except D-Xylose and 4-Hydroxy Benzoic Acid.

After the application of copper hydroxide, the metabolic function of phyllosphere microorganisms in healthy group was opposite to that in diseased group. After 5 days of copper hydroxide application, the metabolism of 31 carbon sources among the microorganisms in the healthy group was inhibited, and there was no effective carbon source. After 10 days of copper hydroxide application, the metabolic function of phyllosphere microorganisms in healthy group gradually recovered, but there was still no efficient carbon source. After 15 days of copper hydroxide application, the metabolic function of phyllosphere microorganisms in healthy group was further restored. The phyllosphere microorganisms in healthy group can efficient metabolism of β-Methyl-D-Glucoside, D-Mannitol, D-Glucosaminic Acid, L-Asparagine, Glycyl-L-Glutamic Acid, the rest of the carbon metabolism is still suppressed.

## Discussion

Microbial communities are sensitive indicator of stress and provides useful data for the study of applied and basic environmental events (Chen T. et al., 2020). Phyllosphere fungi are an important component of the phyllosphere ecosystem, mainly including epiphytic fungi and endophytic fungi, and play an important role in the phyllosphere ecosystem with obvious species diversity and metabolic functions (Arnold et al., 2003; Yao et al., 2019). Bacteria are considered to be the most abundant group in the phyllosphere. These phyllospheric bacteria can be endophytic or epiphytic (Newton et al., 2010), pathogenic and non-pathogenic (Mazinani et al., 2017). External interference (including chemical and physical) can induce responses from microbial communities in phyllosphere ecosystems (Doherty et al., 2017; Karlsson et al., 2017). In addition to acting on target microorganisms, fungicides also tend to affect other non-target microorganisms, thereby altering the composition and structure of microbial communities (Demanou et al., 2006). Our study showed that the effects of copper hydroxide fungicide on community composition and metabolic function of healthy tobacco leaves and diseased tobacco leaves were different.

### Tobacco leaf microbiome prior to copper hydroxide application

Our study showed that Ascomycota and Basidiomycota were the dominant phylum level in both diseased and healthy tobacco leaves in the untreated group. The relative abundance of Ascomycetes was higher in the diseased group than in the healthy group, whereas the relative abundance of Basidiomycetes was lower than that of the healthy group. A study on phyllosphere microbial diversity of boreal forest tree (*Populus balsamifera* L.) also found a similar phenomenon (Bálint et al., 2015). Another study conducted high-throughput sequencing of the fungal communities of 51 tree species in the tropical rain forest showed that the fungal communities on leaves were also dominated by Ascomycota and Basidiomycetes (Kembel and Mueller, 2014). Before the application of fungicide, Proteobacteria and Firmicutes were the dominant levels in both diseased and healthy tobacco leaves, while there were Bacteroidetes and Actinobacteria with low abundance. Many studies have shown that Proteobacteria is the dominant leaf group of various crops, such as lettuce (Rastogi et al., 2012), grape (Singh et al., 2019), Soybean, Shamrock and Arabidopsis (Delmotte et al., 2009), rice (Knief et al., 2012). The Gram-negative bacteria of Proteobacteria can rely on the QS mechanism to ensure that they compete with other bacteria in the Phyllosphere environment of nutrient deficiency (Müller and Ruppel, 2014). This may be the reason why Proteobacteria occupy a favorable niche and are the only dominant bacteria under the pressure of copper hydroxide.

Our data indicated that the most dominant gensera of fungi with healthy leaves prior to treatment with copper hydroxide were *Alternaria* and *Cladosporium*, whereas in diseased leaves it was *Alternaria*. As expected, *Alternaria* is the dominant genus among diseased leaves, as *Alternaria alternata* is the causative agent of tobacco brown spot. However, we also found high relative abundances of *Alternaria*, *Cladosporium*, and *Boeremia* in healthy leaves. *Alternaria* in healthy tobacco leaves may come from diseased tobacco leaves and cause disease once the environmental conditions are suitable. Similar results were reported by Sun et al. (2023) in tobacco target spot. *Cladosporium* contains a number of potential plant pathogens that can cause diseases in a variety of crops such as Lettuce (Hunter et al., 2015), tomato (Rivas and Thomas, 2005; Toju et al., 2019), cucumber (Batta, 2004), canola (Idnurme et al., 2021), tobacco (Wang et al., 2014). *Boeremia* also contains a number of potential plant pathogens that can cause diseases in a variety of crops such as sweet potato (Colmán et al., 2020), Panicle hydrangea (Garibaldi et al., 2018), Walnuts (Wang et al., 2022). However, no pathogenicity has been reported in tobacco. In addition, The study of Chen Y. et al. (2020) showed the potato endophytic fungus *Boeremia* exigua could significantly inhibit the growth of pathogenic fungi of potato late blight.

Our data indicated that the most dominant genera of bacteria with healthy leaves prior to copper hydroxide treatment were *Pantoea* and Weissella, whereas in diseased leaves it was *Pantoea*, *Pseudomonas* and *Sphingomonas*. The results showed that all the dominant genera were Proteobacteria. The study of Lv et al. (2012) showed Proteobacteria gram-negative bacteria can regulate the density of interleaf bacterial community through AHLs to occupy a favorable ecological niche. *Pantoea* was the dominant bacterium in both healthy and infected tobacco leaves before using fungicide, which may be due to its strong adaptability in different environments. Canamas et al. (2007) found *Pantoea* sp. could accumulate glycine-betaine, ectoine and amino acids in the bacterial cells, which enable the *Pantoea* sp. to resist environmental stress.

### Changes in fungal communities after copper hydroxide application

Analysis of alpha diversity in the healthy and diseased groups in this study showed that copper hydroxide treatment increased fungal diversity in both the diseased and healthy groups and there was a significant difference between healthy and diseased leaves at 15 days. Similar results were reported by Peixoto et al. (2021) copper hydroxide can affect soil microbial community and lead to changes in soil microbial composition. The study of Liu et al. (2022) showed that the application of Bordeaux mixture on healthy tobacco increased the diversity of fungal community. This may be due to the selectivity of fungi to copper hydroxide, so that fungicides only inhibit the metabolism of some fungi. The study of Liu et al. (2022) showed that the dominant fungal genera were *Fusarium* and *Cercospora* after spraying Bordeaux mixture on healthy tobacco leaves. After copper hydroxide exposure, the relative abundance of *Alternaria* and *Cladosporium* decreased in both the diseased and healthy groups, while six fungi genera (*Boeremia*, *Issatchenkia*, *Stagonosporopsis*, *Symmetrospora*, *Epicoccum* and *Phoma*) of the top 10 genera were increased. This may be attributed to a decrease in the abundance of pathogenic fungi, leading to the growth of less competitive microorganisms. Copper hydroxide can reduce the relative abundance of *Alternaria* and *Cladosporium* in both healthy and diseased tobacco leaves, which may indicate that copper hydroxide can be used to control tobacco brown spot. Copper hydroxide fungicides had less effect on the microflora structure of diseased leaves than healthy leaves at different times after application. Healthy tobacco leaves have long-lasting protection after the application of fungicides. Therefore, in terms of disease prevention and control, it is recommended that copper preparations be used at the early stage of the disease and when the disease index is low. *Epicoccum* and *Symmetrospora* were unique genera found in tobacco leaf phyllosphere originally infected by *A. alternata*. After the application of copper hydroxide, the relative abundance of *Thanatephorus*, *Epicoccum* and *Phoma* in diseased tobacco leaves did not change much with time, but increased significantly at 10 days in healthy tobacco leaves. This indicates that the prevention of targeted pathogens in healthy tobacco leaves should also pay attention to the prevention of diseases caused by some potential pathogens.

### Changes in bacteria communities after copper hydroxide application

Copper hydroxide treatment increases the relative abundance of Proteobacteria in healthy and diseased tobacco leaves, while decreasing the relative abundance of Firmicutes and Actinobacteria, possibly due to the fact that Firmicutes and Actinobacteria are more sensitive to copper than Proteobacteria. A study on the change of microbial diversity of soil microorganisms under copper stress (Zhang et al., 2020) also found that copper fungicides could change the results of Phyllosphere microbial community, among which Proteobacteria significantly increased and Actinobacteria significantly decreased. After the application of copper hydroxide, the relative abundance of *Pantoea* decreased significantly at 5 days in both diseased and healthy leaves, indicating that *Pantoea* is more sensitive to copper ions. *Pantoea* sp. has a broad range of biological activities, including control of fire blight on fruit trees (Wright et al., 2001), and suppression of several diseases caused by bacteria or fungi on various plants (Braun-Kiewnick et al., 2000; Han et al., 2000; Ortmann and Moerschbacher, 2006). The relative abundance of *Pseudomonas* and *Sphingomonas* in the diseased leaves increased on day 5, which may be due to the plant’s ability to use copper to release growth factors to promote the growth of beneficial bacteria. *Pseudomonas* are primarily considered plant pathogens (Buell et al., 2003; Yu et al., 2013), although some *Pseudomonas* species are known to inhibit leaf fungal pathogens by producing antibiotics (De Meyer and Hofte, 1997). Most research on *Sphingomonas* has focused on the production of growth stimulating factors in plants (Adhikari et al., 2001; Enya et al., 2007; Tsavkelova et al., 2007; Khan et al., 2014). The differences within groups were greater than those between groups, which might be due to the variable leaf surface features, such as the junctions of epidermal cell walls, the grooves of leaf veins, stomata, trichosomes and drainers, which were the priority attachment points for bacteria, leading to the uneven distribution of bacteria on the leaf surface (Müller and Ruppel, 2014). After the application of copper hydroxide, the relative abundance of *Ralstonia* decreased significantly on day 15 in both diseased and healthy leaves, indicating that *Ralstonia* is more sensitive to copper ions. The study of Chen et al. (2019) showed CuONPs significantly reduced bacterial activity and killed *Ralstonia solanacearum* at high concentrations.

### Changes of microbial metabolic capacity after application of copper hydroxide

A variety of compounds such as carbohydrates, amino acids, methanol and other organic compounds are the main sources of carbon and nitrogen for phyllosphere microorganisms. These compounds are often passively leached from the plant interior to 7% of the leaf surface. Although these sources are not abundant, they are sufficient to explain the presence and community structure of microorganisms in the phyllosphere environment (Mercier and Lindow, 2000). The metabolic level of ECO healthy group was significantly reduced after copper hydroxide treatment, which may be due to the characteristics of most phyllosphere microbiome [Vi-able But Nonculturable (VBNC)]. Compared with diseased leaves, healthy leaves have more microbial species, which may result in higher copper stress on metabolic level. Under the influence of copper fungicides, microorganisms will maintain low metabolic activity and last for a long time after entering the VBNC state (Oliver, 2010; Dinu and Bach, 2011). A study by Zhao et al. (2018) showed that copper fungicide could significantly downregulate some intermediates of the tricarboxylic acid (TCA) cycle by 1.4 ~ 2.4 times, indicating that copper could cause carbohydrate metabolism disorder.

## Conclusion

In conclusion, illumina high-throughput sequencing technology and biological microbial analysis techniques were used to analyze the microbial changes in healthy and diseased tobacco leaves after the application of copper hydroxide. The results showed that the application of copper hydroxide fungicide could change the composition of fungal and bacterial communities, and lead to the overall increase of microbial diversity in the phyllosphere. While copper hydroxide decreased the relative abundance of pathogenic bacteria in *Alternaria* and *Cladosporium*, it also decreased the number of beneficial bacteria such as actinomyces and *Pantoea*, and increased the number of potential pathogenic bacteria such as *Boeremia* and *Stagonosporopsis*, *Epicoccum* and *Phoma*.

## Data availability statement

The datasets presented in this study can be found in online repositories. The names of the repository/repositories and accession number(s) can be found in the article/supplementary material.

## Author contributions

HW, FW, and SZ contributed to conception and design of the study. The experiment and data analysis of TL and RF were carried out. RF wrote the first draft of the paper. HW, XC, LC, and SZ made changes to the original manuscript. All authors contributed to the article and approved the submitted version.
